# User-Centric Cell-Free Massive MIMO with Low-Resolution ADCs for Massive Access

**DOI:** 10.3390/s24165088

**Published:** 2024-08-06

**Authors:** Jin-Woo Kim, Hyoung-Do Kim, Kyung-Ho Shin, Sang-Wook Park, Seung-Hwan Seo, Yoon-Ju Choi, Young-Hwan You, Hyoung-Kyu Song

**Affiliations:** 1 Department of Information and Communication Engineering, Sejong University, Seoul 05006, Republic of Korea; kjwccm@naver.com (J.-W.K.); gudeh8330@naver.com (H.-D.K.); shinkh1000@naver.com (K.-H.S.); share1211@naver.com (S.-W.P.); buffalo1997@naver.com (S.-H.S.); jj010513@naver.com (Y.-J.C.); 2 Department of Convergence Engineering for Intelligent Drone, Sejong University, Seoul 05006, Republic of Korea; yhyou@sejong.ac.kr; 3 Department of Computer Engineering, Sejong University, Seoul 05006, Republic of Korea

**Keywords:** massive MIMO, cell-free massive MIMO, fronthaul capacity, low-resolution ADCs, massive access, AP-UE association, user-centric network

## Abstract

This paper proposes a heuristic association algorithm between access points (APs) and user equipment (UE) in user-centric cell-free massive multiple-input-multiple-output (MIMO) systems, specifically targeting scenarios where UEs share the same frequency and time resources. The proposed algorithm prevents overserving APs and ensures the connectivity of all UEs, even when the number of UEs is significantly greater than the number of APs. Additionally, we assume the use of low-resolution analog-to-digital converters (ADCs) to reduce fronthaul capacity. While realistic massive access scenarios, such as those in Internet-of-Things (IoT) environments, often involve hundreds or thousands of UEs per AP using multiple access techniques to allocate different frequency and time resources, our study focuses on scenarios where UEs within each AP cluster share the same frequency and time resources to highlight the impact of pilot contamination in dense network environments. The proposed algorithm is validated through simulations, confirming that it guarantees the connection of all UEs and prevents overserving APs. Furthermore, we analyze the required fronthaul capacity based on quantization bits and confirm that the proposed algorithm outperforms existing algorithms in terms of SE and average SE performance for UEs.

## 1. Introduction

Massive multiple-input-multiple-output (MIMO) is a key technology in fifth-generation (5G) wireless communication systems that support a large number of users simultaneously using a significant number of antennas at an access point (AP) or base station (BS) with the same resources [1]. The channel hardening and favorable propagation characteristics of massive MIMO can increase spectral efficiency (SE) and energy efficiency (EE). With simple linear signal processing techniques at the AP, it can achieve nearly optimal performance [2]. Additionally, massive MIMO has been demonstrated as a promising technology to support massive access. It achieves this by leveraging spatial degrees of freedom, enabling simultaneous transmission by multiple users, as demonstrated in [3]. The cellular structure based on this massive MIMO has been the subject of many studies. However, users located at the cell’s edge experience reduced performance due to their distance from the AP and interference from other cells in the vicinity, leading to performance degradation. There is also an issue of significant performance differences for users located at the center of the cell [4]. Cell-free massive MIMO, unlike traditional cellular systems, involves multiple APs within the coverage area collaborating to support users. This approach promises better performance and is being researched as a key technology for beyond fifth-generation (B5G) and sixth-generation (6G) networks, with the potential to improve the edge effect observed in traditional cellular networks. In particular, cell-free massive MIMO networks can provide wider coverage and are resistant to shadow fading, making them suitable for massive access scenarios [5].

In traditional cell-free massive MIMO, multiple APs and user equipment (UEs) are distributed within the service area, and all UEs communicate with the APs using the same time-frequency resources [6]. All APs are connected to the central processing unit (CPU) through a fronthaul link. This connection allows them to share information. The CPU performs encoding or decoding of the data received from the APs [7]. Traditional cell-free massive MIMO has the advantage of achieving higher SE, EE, and coverage probability compared to conventional cellular systems [8]. However, in traditional cell-free massive MIMO systems, where all APs simultaneously support all UEs, the fronthaul capacity requirements and computational complexity increase significantly as the number of APs and UEs in the network grows [9]. User-centric cell-free massive MIMO, which is an alternative structure to cell-free massive MIMO, involves subsets of APs forming clusters to support UEs. This approach can reduce the load on the fronthaul links compared to traditional cell-free massive MIMO and achieve scalability [10,11,12].

Even in massive access scenarios with a very large number of UEs, user-centric cell-free massive MIMO has the advantage of guaranteeing better performance compared to traditional cellular systems. Additionally, since user-centric cell-free massive MIMO can reduce the fronthaul load, it is more suitable for massive access scenarios than traditional cell-free massive MIMO. However, due to the large number of UEs, some UEs may not be supported by any AP. Also, in user-centric cell-free massive MIMO, channel estimation is performed in the uplink phase using orthogonal pilots among UEs. While massive access scenarios can involve various methods for resource allocation, this study focuses on scenarios where UEs share the same time-frequency resources. This means they utilize the same frequency bands and time slots for communication. Consequently, the limited number of orthogonal pilots becomes a significant issue, as the orthogonal pilots become insufficient to uniquely identify each UE, leading to strong pilot contamination. This contamination occurs when multiple UEs are assigned the same pilot sequence, causing interference during channel estimation. Such interference severely degrades the spectral efficiency (SE) performance in user-centric cell-free massive MIMO systems [7,13]. Therefore, to minimize severe pilot contamination and achieve load balancing for the APs, each AP has a limit on the number of UEs it can support [12].

Cell-free massive MIMO, in which a large number of APs with one or multiple antennas support UEs coherently, can ensure high SE and uniformly good service to UEs [14]. However, in cell-free massive MIMO systems with a large number of APs, the number of radio frequency (RF) chains also increases. The high-resolution analog-to-digital converters (ADCs) in the RF chains become impractical due to high power consumption and significant hardware costs [15,16,17,18]. Additionally, it is necessary to consider the impact of hardware impairments to account for realistic environments [19,20]. As the number of users increases, the required fronthaul capacity also increases. Therefore, in massive access scenarios, it is essential to reduce the fronthaul capacity.

### 1.1. Motivation

To configure a user-centric cell-free massive MIMO system, each UE should select an AP subset that can ensure good performance while pilot contamination is minimized. However, the problem of ensuring the connection of all users within the coverage area while simultaneously limiting the number of UEs serviced by an AP is NP-hard and has very high computational complexity [10]. Therefore, a heuristic approach is more suitable [10]. The AP-UE association process can take various forms. These include each AP individually selecting the UEs that it directly supports. Each UE selects the AP subset that supports it. Additionally, there is a combined approach that incorporates both methods. In [21], the association process involves each AP comparing the large-scale fading coefficients (LSFCs) of all UEs it serves. Each AP calculates the LSFC for all UEs and then supports them in order of highest LSFC. To avoid strong pilot contamination, the number of users that can be supported is limited. However, because the association is formed by the AP, in situations with a large number of UEs, UEs with relatively low LSFC values may risk losing connectivity. In particular, in environments with a high number of UEs, if the number of UEs that an AP can support is fixed, such unfair access will become more prevalent. Therefore, there is a need to provide a method that can guarantee connectivity for all UEs. In [12], each AP is limited to supporting a number of UEs equal to the length of the orthogonal pilot. Initially, each UE selects the AP with the largest LSFC as its master AP. Then, for each pilot, each AP supports the UE with the best channel condition. This approach proposes a scalable AP-UE association by ensuring connectivity for all UEs while the number of UEs connected to each AP is limited. However, this method assumes that if the number of UEs per AP exceeds the number of pilots, multiple UEs can be assigned to the same pilot but are multiplexed in time and/or frequency resources to avoid pilot contamination, as described in [12]. While this approach can mitigate pilot contamination, it relies on multiple access techniques that allocate different time or frequency resources to each UE. In [22], the process involves sorting UEs based on LSFC for each AP. Then, it sequentially connects a number of UEs equal to the length of the orthogonal pilot and prioritizes those with higher LSFC. This process repeats until all APs are fully connected. In this process, unconnected UEs will request a connection to the AP with the highest LSFC. While this allows all UEs to be connected, in environments with a large number of UEs, there will likely be an increase in overserving APs that suffer from significant pilot contamination. This will prevent the system from guaranteeing good performance. In [23], it is assumed that each UE knows the LSFC with all APs. The clustering is then conducted by connecting to APs with LSFC values exceeding a certain threshold. Similar to [22], this approach ensures connectivity for all UEs, but this leads to an imbalance in the load among APs. Additionally, the above studies do not consider methods to reduce fronthaul capacity as the number of users increases in massive access scenarios.

In practical cell-free massive MIMO systems, the fronthaul capacity is not infinite. Additionally, the large number of APs in these systems leads to increased power consumption when high-resolution ADCs are used. On the other hand, low-resolution ADCs can mitigate this issue by reducing power consumption and hardware costs, as well as decreasing the fronthaul load between the APs and the CPU. Consequently, research works on the application of low-resolution ADCs in cellular and cell-free massive MIMO systems are actively being conducted. In [24], the use of low-resolution ADCs in wireless communication systems is surveyed, emphasizing their benefits in reducing power consumption and hardware complexity. Despite introducing quantization noise, these ADCs can maintain system performance through techniques such as larger antenna arrays and advanced signal processing. In [25], a framework for cell-free mmWave massive MIMO systems is presented. This study shows how low-resolution ADCs, combined with hybrid precoding/decoding, can improve performance, reduce power consumption, and handle limited fronthaul capacity. Additionally, ref. [26] underscores the importance of efficient fronthaul usage and advanced signal processing techniques in maintaining high performance in cell-free massive MIMO systems equipped with low-resolution ADCs. This study highlights how these techniques contribute to reducing overall power consumption. In [27], the uplink performance of cell-free massive MIMO systems using low-resolution ADCs at access points is analyzed, revealing that 5-bit ADCs strike a balance between minimizing hardware cost and power consumption while sustaining adequate system performance. Finally, ref. [15] explores the uplink performance of cell-free massive MIMO systems under conditions of limited fronthaul capacity and hardware impairments. This study emphasizes the critical role of reducing power consumption through efficient ADC solutions to maintain system performance.

### 1.2. Contribution

As mentioned in the above research works, in user-centric cell-free massive MIMO systems, AP-UE association can lead to load imbalance among APs or result in low-LSFC UEs not being connected. Even when load balancing is implemented by limiting the number of UEs connected to each AP to ensure connectivity for all UEs, there is still a lack of consideration for reducing the required fronthaul capacity in environments with a large number of UEs. Therefore, in this study, a heuristic-based AP-UE association method is proposed for user-centric cell-free massive MIMO systems using low-resolution ADCs. The association utilizes LSFC between APs and UEs. It takes into account imperfect channel state information (CSI). Additionally, this paper assumes that APs are equipped with low-resolution ADCs to reduce fronthaul capacity in massive access scenarios while considering practical conditions. Research on cellular and cell-free massive MIMO systems using low-resolution ADCs is actively underway because low-resolution ADCs can reduce the exponentially increasing power consumption and costs associated with resolution. Additionally, it can also reduce the fronthaul load between APs and CPUs [15,16,17,25,26,27]. The main contributions of this study are as follows:

- Since we assume a user-centric cell-free massive MIMO environment with limited fronthaul capacity and a very large number of UEs, we consider the deployment of APs equipped with low-resolution ADCs. The quantization noise model due to low-resolution ADCs is described as the additive quantization noise model (AQNM), as depicted in [26]. Additionally, we derive closed-form expressions for uplink SE in systems using low-resolution ADCs.
- Although there is existing research on user-centric cell-free massive MIMO, there are few studies that conduct simulations in massive access scenarios where the number of UEs exceeds the number of APs. Some studies propose association algorithms that can accommodate a large number of UEs, but in environments with a very high number of UEs, these approaches often result in overserving APs or fail to guarantee UE connectivity. Overserving APs, which lead to pilot contamination, significantly degrade SE performance. In response, we propose an AP-UE association algorithm that prevents overserving APs and ensures the connectivity of all UEs in massive access scenarios.
- This study specifically focuses on scenarios where UEs share the same time-frequency resources, which is critical for understanding the impact of pilot contamination in dense network environments. Unlike traditional approaches that rely on multiple access techniques, our method manages pilot contamination and maintains connectivity and performance within the constraints of shared resources.
- In a user-centric cell-free massive MIMO system, the association problem, which guarantees connectivity for all UEs while the number of UEs supported by each AP is limited, is NP-hard and computationally intensive. Therefore, in this study, we propose an association algorithm using a heuristic approach to address this challenge.
- The proposed approach not only enhances connectivity and performance in current networks but also lays the groundwork for scalable solutions in future large-scale Internet-of-Things (IoT) and 6G deployments, where efficient resource management and reduced fronthaul load are critical.

### 1.3. Organization and Notations

The remainder of this paper is organized as follows. In Section 2, we introduce the system model of user-centric cell-free massive MIMO with fronthaul capacity constraints. In Section 3, we derive the closed-form expressions for uplink SE. Section 4 proposes the AP-UE association algorithm, while Section 5 analyzes the simulation results. Finally, in Section 6, we conclude our study.

Notation: In boldface, lowercase x represents a vector, while uppercase X represents a matrix. $X_{i,j}$ denotes the entry i,j, and the superscripts ^−1^, ^*T*^, *, and ^*H*^ denote inverse, transpose, conjugate, and conjugate transpose, respectively. The *N*-dimensional identity matrix is denoted by $I_{N}$ and the *N*-dimensional zero matrix is denoted by $0_{N}$. The symbol E represents the expectation operator. $R_{xy}$ denotes the correlation matrix between x and y. The symbol C denotes the set of all complex numbers, and the symbol CN(0,1) denotes the complex Gaussian distribution with a mean of 0 and a variance of 1.

## 2. System Model

**Figure 1:**
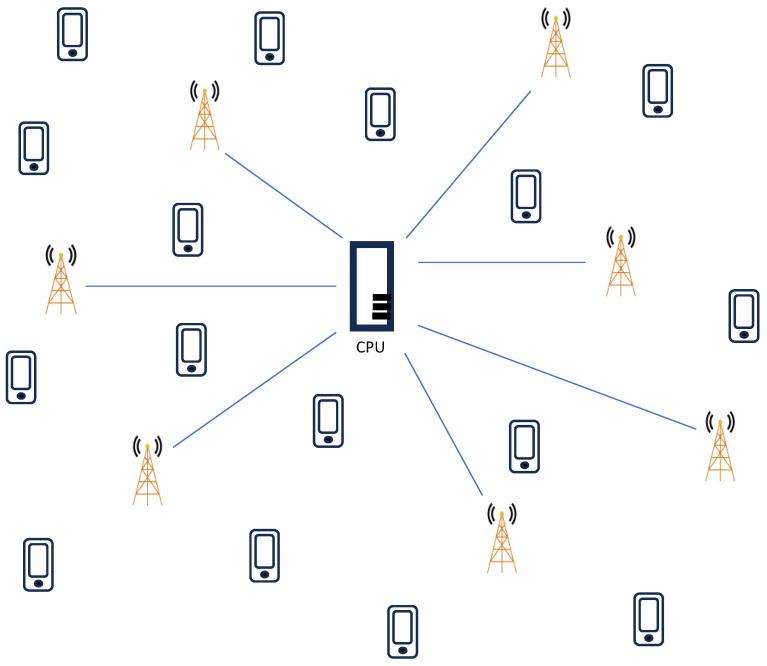
Cell-free massive MIMO system model.

In this study, we consider only the uplink phase of a user-centric cell-free massive MIMO system with limited fronthaul capacity, in which *L* APs equipped with *N* antennas are distributed across the service area, and *K* UEs equipped with single antennas are present. As illustrated in Figure 1, all APs are connected to a single CPU via fronthaul links, and the APs form subsets to support UEs in a user-centric manner. Additionally, all APs employ a low-resolution ADC architecture.

We assume that the number of UEs each AP can support is limited by the number of orthogonal pilots, $\tau_{p}$. Specifically, we consider scenarios where $L\ll K\leq L\times \tau_{p}$. This limitation ensures that each AP can support up to $\tau_{p}$ UEs without causing overserving APs and severe pilot contamination.

We acknowledge that realistic massive access scenarios, such as those in IoT environments, often involve hundreds or thousands of UEs per AP. These scenarios use multiple access techniques to allocate different frequency and time resources to each UE. However, our study focuses on scenarios where UEs share the same frequency and time resources. By doing so, we aim to highlight the impact of pilot contamination in dense network environments where multiple access techniques are not employed.

**Remark** **1.** *In practical deployments, a combination of multiple access techniques and increased AP density would be necessary to support a larger number of UEs. Multiple access techniques such as time division multiple access (TDMA) or orthogonal frequency division multiple access (OFDMA) can be used to allocate different time/frequency resources to UEs, thus accommodating more UEs per AP without causing pilot contamination. Additionally, increasing the number of APs proportionally can help maintain system performance in dense environments. While our current study focuses on the constraints imposed by orthogonal pilots, we recognize the need for realistic scenarios where the number of UEs may exceed $L\times \tau_{p}$. In such cases, network operators would typically deploy more APs or employ multiple access techniques to handle the increased load, ensuring the proposed algorithm can still function effectively.*

### 2.1. Channel Model and Quantization Model

Because the antennas have non-uniform radiation patterns and the physical propagation environment affects certain spatial directions, the channels are generally spatially correlated [2,28]. Therefore, the channel in this paper utilizes the correlated Rayleigh block fading model used in [2]. The channel between the AP *l* and the UE *k* is represented as follows:

(1) $$g_{kl}=\sqrt{\beta_{kl}}h_{kl},$$

where $\beta_{kl}$ represents the large-scale fading factor, which includes path loss and shadowing effects, and $h_{kl}\in C^{N}$ is an independent and identically distributed (i.i.d.) small-scale fading variable with the CN(0,1) distribution. Under the assumption of block fading channels, it is assumed that the CSI remains constant during coherence intervals $\tau_{c}$. Among these intervals, a portion of duration $\tau_{p}$ is allocated for uplink training, while the remainder $\tau_{c}-\tau_{p}=\tau_{u}$ is allocated for uplink data transmission.

Unlike conventional cell-free massive MIMO research that uses high-resolution or perfect-resolution ADCs, this study assumes user-centric massive MIMO that considers fronthaul capacity constraints and employs low-resolution ADCs. In the signal transmission process, APs and CPUs that use low-resolution ADCs introduce the issue of nonlinear compression of signals [26]. We refer to $Q_{\alpha }\left(y\right)$ as the mathematical representation of the generic scalar quantizer result for signal $y\in C^{N}$. The classical approach dealing with such nonlinear characteristics uses Bussgang decomposition as follows [29]:

(2) $$\tilde{y}=Q_{\alpha }\left(y\right)=F_{\alpha }y+q_{\alpha },$$

where $F_{\alpha }\in C^{N\times N}$ can be obtained from the linear minimum mean square error (MMSE) estimation of $\tilde{y}$ as follows:

(3) $$F_{\alpha }=E\left\{\tilde{y}y\right\}{\left(E\left\{yy^{H}\right\}\right)}^{-1}=R_{\tilde{y}y}R_{yy}^{-1},$$

and the quantization noise $q_{\alpha }\in C^{N}$ has the correlation matrix as follows:

(4) $$\begin{array}{cc} R_{q_{\alpha }q_{\alpha }} & =E\left\{\left(\tilde{y}-F_{\alpha }y\right)\right\}\\ & =R_{\tilde{y}\tilde{y}}-R_{\tilde{y}y}F_{\alpha }^{H}-FR_{y\tilde{y}}+FR_{yy}F_{\alpha }^{H}\\ \; & =R_{\tilde{y}\tilde{y}}-R_{\tilde{y}y}R_{yy}^{-1}R_{y\tilde{y}}. \end{array}$$

Generic scalar quantizers approximate the covariance matrix through approximation methods. In [30], the covariance matrix of such quantizers was approximated using the distortion factor $\rho_{\alpha }=\left(1-\alpha \right)=1/{\mathrm{SQNR}}_{\alpha }$ based on the signal-to-quantization noise ratio (SQNR). In [30], $q_{\alpha }$ can be approximated as follows:

(5) $$R_{\tilde{y}y}=R_{y\tilde{y}}=\alpha R_{yy},$$

and

(6) $$R_{\tilde{y}\tilde{y}}\approx \alpha^{2}R_{yy}+\alpha \left(1-\alpha \right)\mathrm{diag}\left(R_{yy}\right).$$

By using the Equations (5) and (6), we can approximate the Bussgang decomposition of Equation (2) as follows:

(7) $$\tilde{y}=Q_{\alpha }\left(y\right)\approx \alpha y+{\hat{q}}_{\alpha },$$

where ${\hat{q}}_{\alpha }∼\mathrm{CN}\left(0,R_{{\hat{q}}_{\alpha }{\hat{q}}_{\alpha }}\right)\in C^{N}$ and the correlation matrix as follows:

(8) $$R_{{\hat{q}}_{\alpha }{\hat{q}}_{\alpha }}=\alpha \left(1-\alpha \right)\mathrm{diag}\left(R_{yy}\right).$$

This approximation can effectively approximate quantization noise, but it has limitations in that it approximates nonlinear characteristics with linear ones.

In [31], the optimal distortion factor $\rho_{\alpha }$ of uniform and non-uniform quantizers according to the number of bits was tabulated. The distortion parameters are expressed in Table 1

### 2.2. Uplink Training Phase

During the coherence interval $\tau_{c}$, UEs transmit pilot sequences to the APs for channel estimation. Additionally, because it is assumed to be in TDD mode, only channel state information (CSI) is required through the uplink training phase. The orthogonal pilot signals of duration $\tau_{p}$ are denoted as $\phi_{1},\ldots ,\phi_{\tau_{p}}$, which all pilot signals satisfy ${∥\phi_{t}∥}^{2}=1$. Since we assume a massive access scenario with a large number of UEs, we consider $K>\tau_{p}$, which means the orthogonal pilot length $\tau_{p}$ is less than the number of UEs. Therefore, some UEs should share the same pilot sequence. The set of pilot indices is denoted by $i_{k}\in 1,\ldots ,\tau_{p}$, and the set of UEs that share the same pilot with UE *k* and include UE *k* is denoted as $D_{k}$.

The received signal $y_{i_{k},l}^{p}\in C^{N}$ at AP *l* from the pilot signal $\phi_{i_{k}}$ with index $i_{k}$ is given as follows:

(9) $$y_{i_{k},l}^{p}=\sum_{k\in D_{k}}\sqrt{\tau_{p}\rho_{k}}g_{kl}+n_{i_{k},l},$$

where $\rho_{k}$ denotes the pilot transmission power of UE *k*, and $n_{i_{k},l}∼\mathrm{CN}\left(0,\sigma^{2}I_{N}\right)\in C^{N}$ denotes the thermal noise. Also, the received pilot signal at the AP is quantized before transmission to the CPU, (9) is expressed as follows:

(10) $$\begin{array}{cc} {\tilde{y}}_{i_{k},l}^{P} & =Q_{\alpha }\left(y_{i_{k},l}^{p}\right),\\ \; & \approx \alpha \left(\sum_{k\in D_{k}}\sqrt{\tau_{p}\rho_{k}}g_{kl}+n_{i_{k},l}\right)+{\hat{q}}_{\alpha }. \end{array}$$

If the MMSE estimation of the channel employed in [2] is used, the estimated channel is as follows:

(11) $${\hat{g}}_{kl}=\alpha \sqrt{\tau_{p}\rho_{k}}R_{kl}\Psi_{i_{k}l}^{-1}{\tilde{y}}_{i_{k},l}^{P}.$$

where the correlation matrix of (10) is as follows:

(12) $$\Psi_{i_{k}l}=E\left\{{\tilde{y}}_{i_{k},l}^{P}{\left({\tilde{y}}_{i_{k},l}^{P}\right)}^{H}\right\}=\sum_{k\in D_{k}}\alpha^{2}\rho_{k}\tau_{p}R_{kl}+\sigma^{2}I_{N}+R_{{\hat{q}}_{\alpha }{\hat{q}}_{\alpha }}\in C^{N\times N}.$$

Therefore, the channel estimate is distributed as follows:

(13) $${\hat{g}}_{kl}∼\mathrm{CN}\left(0,\alpha^{2}\rho_{k}\tau_{p}R_{kl}\Psi_{i_{k}l}^{-1}R_{kl}\right)\in C^{N}.$$

And estimation error, ${\tilde{g}}_{kl}=g_{kl}-{\hat{g}}_{kl}$, is independent with ${\hat{g}}_{kl}$ and is distributed as follows:

(14) $${\tilde{g}}_{kl}∼\mathrm{CN}\left(0,R_{kl}-\alpha^{2}\rho_{k}\tau_{p}R_{kl}\Psi_{i_{k}l}^{-1}R_{kl}\right)\in C^{N}.$$

### 2.3. Uplink Data Transmission Phase

During the data transmission phase, the APs receive uplink data from all UEs. The signal received by AP *l* from all UEs can be expressed as follows:

(15) $$y_{l}=\sum_{k=1}^{K}g_{kl}s_{k}+n_{l}\in C^{N},$$

where $s_{k}∼\mathrm{CN}\left(0,p_{k}\right)$ represents the received signal transmitted from UE *k*, which the power is $p_{k}$, and $n_{l}∼\mathrm{CN}\left(0,\sigma^{2}I_{N}\right)\in C^{N}$ denotes the received noise.

In this study, it is assumed that local computational tasks are performed at the AP level to avoid CPU overload due to the assumption of a large-scale network. Furthermore, under the assumption of a user-centric cell-free massive MIMO system, each AP supports subsets of UEs. Therefore, in this study, the association between APs and UEs is expressed as follows:

(16) $$M_{k}=\left\{l:S_{kl}=1,l\in \left\{1,\ldots ,L\right\}\right\},$$

and

(17) $$A_{l}=\left\{k:S_{kl}=1,k\in \left\{1,\ldots ,K\right\}\right\},$$

where $M_{k}$ represents the subset of APs supporting UE *k*, and $A_{l}$ represents the subset of UEs supported by AP *l*. And the matrix $S\in R^{L\times K}$ denotes the connectivity between APs and UEs in the user-centric cell-free massive MIMO system. Specifically, when UE *k* is linked to AP *l*, the corresponding entry $S_{kl}$ is equal to 1; otherwise, it remains 0. Furthermore, we define the block-diagonal matrix $D_{k}=\mathrm{diag}\left(D_{k}^{1},\ldots ,D_{k}^{L}\right)$, where the diagonal matrix $D_{k}^{l}\in C^{N\times N}$ represents the antenna configuration between AP *l* and UE *k*. The diagonal elements of $D_{k}^{l}$ indicate whether the antennas of AP *l* are serving UE *k*. Specifically, if AP *l* is serving UE *k*, the diagonal elements are set to 1 (forming an identity matrix $I_{N}$), indicating that all *N* antennas are used to serve this UE. Otherwise, the diagonal elements are set to 0 (forming a zero matrix $0_{N}$).

Furthermore, to reduce the load on the fronthaul link, APs use low-resolution ADCs to send compressed data to the CPU. Therefore, Equation (15) can be quantized and expressed as follows:

(18) $${\tilde{y}}_{l}=\alpha \left(\sum_{k=1}^{K}g_{kl}s_{k}+n_{l}\right)+n_{q}\in C^{N},$$

where $n_{q}\in C^{N}$ represents Gaussian quantization noise, and the covariance matrix is expressed as follows:

(19) $$R_{n_{q}}=\alpha \left(1-\alpha \right)\mathrm{diag}\left(\sum_{k=1}^{K}\rho_{k}R_{kl}+\sigma^{2}I_{N}\right)\in C^{N\times N}.$$

Therefore, each AP can obtain a local estimation for the signal $s_{k}$, as described in [21], as follows:

(20) $$\begin{array}{cc} {\tilde{s}}_{kl} & =v_{kl}^{H}D_{k}^{l}{\tilde{y}}_{l}\\ \; & =\alpha v_{kl}^{H}D_{k}^{l}g_{kl}s_{k}+\alpha v_{kl}^{H}D_{k}^{l}\sum_{j=1,j\neq k}^{K}g_{jl}s_{j}+\alpha v_{kl}^{H}D_{k}^{l}n_{l}+v_{kl}^{H}D_{k}^{l}n_{q}, \end{array}$$

where $v_{kl}\in C^{N}$ represents the combining vector chosen at AP *l* for UE *k*, and the above equation applies to any combining vector [21]. In [32], the maximum ratio (MR) combining with vector $v_{kl}^{MR}=g_{kl}^{H}$ was considered. Also, the local partial MMSE (LP-MMSE) with perfect resolution ADCs at the APs was investigated in [21].

(21) $$v_{kl}=\rho_{k}{\left(\sum_{j\in A_{l}}\rho_{k}\left({\hat{g}}_{jl}{\hat{g}}_{jl}^{H}+C_{jl}\right)+\sigma^{2}I_{N}\right)}^{-1}{\hat{g}}_{kl}$$

Furthermore, the local MMSE (L-MMSE) combining with low-resolution ADCs at the APs was considered in [14].

(22) $$v_{kl}=\alpha \rho_{k}{\left(\alpha^{2}\sum_{j=1}^{K}\rho_{k}\left({\hat{g}}_{jl}{\hat{g}}_{jl}^{H}+C_{jl}\right)+\alpha^{2}\sigma^{2}I_{N}+R_{n_{q}}\right)}^{-1}{\hat{g}}_{kl}$$

However, Equation (21) does not consider the low-resolution ADCs in the combining vector, and Equation (22) is not a scalable combining vector. By utilizing both equations, we can derive the LP-MMSE combining factor using low-resolution ADCs as follows:

(23) $$v_{kl}=\alpha \rho_{k}{\left(\alpha^{2}\sum_{j\in A_{l}}\rho_{k}\left({\hat{g}}_{jl}{\hat{g}}_{jl}^{H}+C_{jl}\right)+\alpha^{2}\sigma^{2}I_{N}+R_{n_{q}}\right)}^{-1}{\hat{g}}_{kl}.$$

Subsequently, the local estimate ${\tilde{s}}_{kl}$ is conveyed to the CPU via the fronthaul link. The CPU then aggregates ${\tilde{s}}_{kl}$ received from all APs using weights $w_{kl}$ to obtain ${\hat{s}}_{k}=\sum_{l=1}^{L}w_{kl}^{*}{\tilde{s}}_{kl}$, which is eventually used to decode $s_{k}$. From Equation (20), we finally obtain the estimate of $s_{k}$, as follows:

(24) $${\hat{s}}_{k}=\alpha v_{k}^{H}W_{k}^{H}D_{k}g_{k}s_{k}+\alpha v_{k}^{H}W_{k}^{H}D_{k}\sum_{j=1,j\neq k}^{K}g_{j}s_{j}+\alpha v_{k}^{H}W_{k}^{H}D_{k}n_{l}+v_{k}^{H}W_{k}^{H}D_{k}n_{q},$$

where $W_{k}^{H}=\mathrm{diag}\left(w_{k1}I_{N},\ldots ,w_{kL}I_{N}\right)\in C^{\left(LN\times LN\right)}$ and $v_{k}\in C^{LN}$. This approach is known as large-scale fading decoding (LSFD) in cellular massive MIMO systems [33,34].

## 3. Spectral Efficiency

In this section, we derive the closed-form SE expression for user-centric cell-free massive MIMO with low-resolution ADCs. Since the CPU does not have knowledge of channel estimation, we utilize the use-and-then-forget (UatF) bound to derive the achievable SE [2].

The process of deriving achievable SE is similar to [2], but the received signal is given by (24). In particular, the first term of (24) is treated as the desired signal, while the second, third, and fourth terms are treated as effective noise. In fact, the signals received from other UEs, as well as quantization and thermal noise, are uncorrelated with the desired signal and also uncorrelated among the effective noise terms [2,7,26].

**Lemma** **1.** *The achievable spectral efficiency for the kth user in a user-centric massive MIMO with low-resolution ADCs is as follows:*

(25) $${\mathrm{SE}}_{k}=\left(1-\frac{\tau_{p}}{\tau_{c}}\right){log}_{2}\left(1+{\mathrm{SINR}}_{k}\right),$$

*where ${\mathrm{SINR}}_{k}$ is given as follows:*

(26) $${\mathrm{SINR}}_{k}=\frac{\alpha^{2}\rho_{k}{\left|E\left\{v_{k}^{H}W_{k}^{H}D_{k}^{H}g_{k}\right\}\right|}^{2}}{\alpha^{2}\sum_{j=1}^{K}\rho_{j}\Gamma_{ik}^{\left(2\right)}-\alpha^{2}\rho_{k}{\left|\Gamma_{k}^{\left(1\right)}\right|}^{2}+\alpha^{2}\sigma^{2}\Gamma_{k}^{\left(3\right)}+\Gamma_{k}^{\left(4\right)}},$$

*where*

(27) $$\Gamma_{k}^{\left(1\right)}=E\left\{v_{k}^{H}W_{k}^{H}D_{k}^{H}g_{k}\right\}$$

(28) $$\Gamma_{ik}^{\left(2\right)}=E\left\{{\left|v_{k}^{H}W_{k}^{H}D_{k}g_{j}\right|}^{2}\right\}$$

(29) $$\Gamma_{k}^{\left(3\right)}=E\left\{{∥D_{k}W_{k}^{H}v_{k}∥}^{2}\right\}$$

(30) $$\Gamma_{k}^{\left(4\right)}=E\left\{{\left|v_{k}^{H}W_{k}^{H}D_{k}{\bar{n}}_{q}\right|}^{2}\right\}.$$

*The ${\mathrm{SINR}}_{k}$ can be simplified as follows:*

(31) $${\mathrm{SINR}}_{k}=\frac{\alpha^{2}\rho_{k}{\left|w_{k}^{H}f_{k}\right|}^{2}}{w_{k}^{H}\left(\alpha^{2}\sum_{j=1}^{K}\rho_{j}\Lambda_{ki}^{\left(1\right)}-\alpha^{2}\rho_{k}f_{k}f_{k}^{H}+\alpha^{2}\sigma^{2}\Lambda_{k}^{\left(2\right)}+\Lambda_{k}^{\left(3\right)}\right)w_{k}},$$

*where*

(32) $$\begin{array}{cc} \; & w_{k}={\left[w_{k1},\ldots ,w_{kL}\right]}^{T}\\ \; & f_{k}={\left[E\left\{v_{k1}^{H}D_{k1}g_{k1}\right\},\ldots ,E\left\{v_{kL}^{H}D_{kL}g_{kL}\right\}\right]}^{T}\\ \; & \Lambda_{ki}^{\left(1\right)}=\left[E\left\{v_{kl}^{H}D_{kl}g_{jl}g_{jm}^{H}D_{km}v_{km}\right\}:l,m=1,\ldots ,L\right]\\ \; & \Lambda_{k}^{\left(2\right)}=\mathrm{diag}\left(E\left\{{∥D_{k1}v_{k1}∥}^{2}\right\},\ldots ,E\left\{{∥D_{kL}v_{kL}∥}^{2}\right\}\right)\\ \; & \Lambda_{k}^{\left(3\right)}=\left[E\left\{v_{kl}^{H}D_{kl}{\bar{n}}_{jl}{\bar{n}}_{jm}^{H}D_{km}v_{km}\right\}:l,m=1,\ldots ,L\right]. \end{array}$$

**Proof of Lemma** **1.** It follows the similar approach as in ([2], Th. 4.4), but for the received signal in (24).    □

The structure of Equation (31) represents the generalized Rayleigh quotient with respect to $w_{k}$. Therefore, the maximum value of ${\mathrm{SINR}}_{k}$ is achieved as follows ([2], Lem. B.10):

(33) $${\mathrm{SINR}}_{k}=\alpha^{2}\rho_{k}f_{k}^{H}{\left(\alpha^{2}\sum_{j=1}^{K}\rho_{j}\Lambda_{ki}^{\left(1\right)}-\alpha^{2}\rho_{k}f_{k}f_{k}^{H}+\alpha^{2}\sigma^{2}\Lambda_{k}^{\left(2\right)}+\Lambda_{k}^{\left(3\right)}\right)}^{-1}f_{k}.$$

The optimal LSFD weight is as follows:

(34) $$w_{k}^{\mathrm{LSFD}}={\left(\alpha^{2}\sum_{j=1}^{K}\rho_{j}\Lambda_{ki}^{\left(1\right)}-\alpha^{2}\rho_{k}f_{k}f_{k}^{H}+\alpha^{2}\sigma^{2}\Lambda_{k}^{\left(2\right)}+\Lambda_{k}^{\left(3\right)}\right)}^{-1}f_{k}.$$

## 4. AP-UE Association Algorithm

In this section, we propose an AP-UE association scheme for user-centric cell-free massive MIMO systems in the context of massive access scenarios in which the number of UEs in the network is significantly greater than the number of APs. When each UE connects to the network, it selects the APs that will support it. To avoid significant pilot contamination, each AP has a limited capacity to support UEs. Also, we apply the following assumptions.

**Assumption** **1.** *Each AP serves at most one UE per pilot and uses all N antennas to serve these UEs.*

(35) $$D_{k}^{l}=\left\{\begin{array}{c} I_{N},\;if\;k\in A_{l}\\ 0_{N},\;if\;otherwise \end{array}\right.$$

*for l = 1, …, L.*

**Assumption** **2.** *In scenarios where the number of UEs exceeds $L\times \tau_{p}$, it is assumed that the CPU allocates different time or frequency resources to APs to accommodate the additional UEs. Each allocated resource operates independently, allowing APs to manage their associated UEs without interference from APs operating on different resources. Consequently, within each allocated resource, the algorithm operates under the assumption that the number of UEs does not exceed $L\times \tau_{p}$, ensuring optimal performance and minimizing pilot contamination.*

In this study, we satisfy Assumptions 1 and 2, and propose an AP-UE association scheme using a heuristic approach. We consider a scenario where all UEs in the network are connected to at least one AP, and the number of UEs exceeds the number of APs. Each AP has a limited capacity to support UEs, so both AP-preference and UE-preference associations need to be considered to ensure that all UEs are connected to at least one AP.

The AP-UE association is conducted using LSFC between APs and UEs, enabling connections to APs with higher LSFC. Due to the large number of UEs, there may be some UEs connected to only one AP. These UEs are associated with the APs that provide the largest LSFC possible.

Due to Assumption 2, the algorithm effectively operates within the constraint of each resource, making it a solution for AP-UE connections within the limit of $L\times \tau_{p}$ UEs per resource. The association process operates through the following steps:

Step 1. During the initial access phase, each UE calculates its LSFC with each AP and selects the AP with the highest LSFC as its master AP, denoted as $l_{m}$, for connection. Also, all APs have a set of UEs denoted as $\left\{A_{l_{m}}^{M}\right\}$, which have selected them as the master AP. As there are more UEs than APs, APs may receive more connection requests than they can support. APs with more connected UEs that they can support exclude the UE with the smallest LSFC from their connections. The excluded UE is included in the exclusion-list, denoted as $E_{k}$, of that AP. And the excluded UE selects the AP with the highest LSFC among the other APs as its master AP.

If the number of UEs exceeds $L\times \tau_{p}$, the CPU dynamically allocates different time or frequency resources to APs to accommodate the additional UEs. Each allocated resource operates independently, allowing APs to manage their associated UEs without interference from APs operating on different resources. Consequently, within each allocated resource, the algorithm operates under the assumption that the number of UEs does not exceed $L\times \tau_{p}$, ensuring optimal performance and minimizing pilot contamination.

This process continues until there are no over-serving APs. Through this method, connectivity is guaranteed for UEs with APs that can provide good performance, while also preventing any AP from becoming overserved during the initial access process.

Step 2. All UEs maintain a list of available AP candidates that exclude the $A^{m}$, $\left\{M_{k}=\emptyset :k=1,\ldots ,K\right\}$ and $E_{k}$, which comprises APs capable of supporting them. And the list of AP candidates is represented as $C_{k}$. $L_{UE}$ represents the index list of UEs, and the connections between APs and UEs are established sequentially based on the $L_{UE}$. UE $k\in \left\{1,\ldots ,K\right\}$ is connected to the AP in $C_{k}$ with the highest LSFC.

(36) $$l=\arg\max_{j\in C_{k}}\beta_{kj}$$

If $\left|A_{l}\right|<\tau_{p}$, UE *k* takes AP *l* as its serving AP by $M_{k}\cup \left\{l\right\}$, and repeats Step 2 to seek for more APs.

Step 3. If the number of UEs supported by AP *l* exceeds $\tau_{p}$, $\left|A_{l}\right|>\tau_{p}$, the LSFCs of the UEs supported by AP *l* are compared. At this point, the UEs that have selected AP *l* as the master AP are excluded from the comparison. The connection of the UE with the smallest LSFC among the compared UEs is terminated, and this UE is included in its $E_{k}$ by AP *l*.

(37) $$k^{\prime }=\arg\min_{i\in A_{l}\setminus A_{l}^{M}}\beta_{il}$$

Step 4. The above process repeats for all UEs until $C_{k}=\emptyset$. The pseudocode of this algorithm is given in Algorithm 1.
**Algorithm 1:** Proposed AP-UE association algorithm
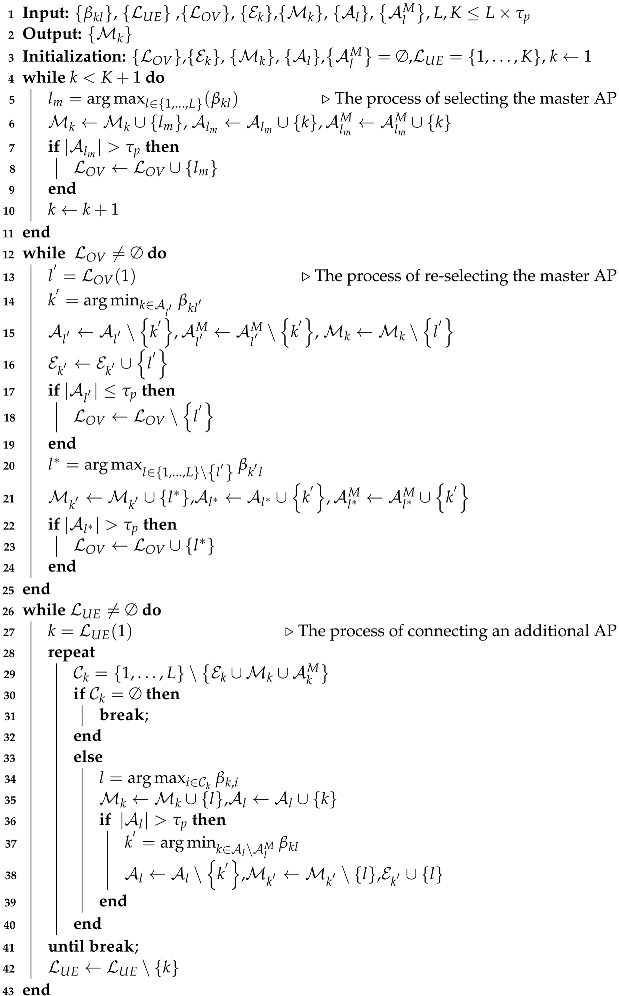


## 5. Simulation Results

### 5.1. Simulation Setting

In this section, we validate the closed-form SE expression proposed in Lemma 1 for user-centric cell-free massive MIMO systems under massive access scenarios. In this paper, we consider a scenario with L=50 APs, in which all APs and UEs are randomly distributed within a square coverage area of 0.5km×0.5km. Furthermore, we assume that all APs are equipped with half-wavelength-spaced uniform linear arrays with N=4. We apply the wrap-around technique to approximate an infinitely large network with 800 antennas$/{\mathrm{km}}^{2}$. The AP-UE association algorithm proposed in Section 4 is applied, and the pilot assignment algorithm is performed using the UE-group method as employed in [21]. This method minimizes pilot contamination by grouping UEs with minimal overlapping APs into the same pilot group. Each UE is assigned a single orthogonal pilot sequence for all its AP connections, ensuring simplicity and coherence in channel estimation. By minimizing the intersection of APs among UEs within the same pilot group, the user-group method effectively reduces pilot contamination, maintaining system performance even in massive access scenarios. To obtain large-scale propagation conditions, we utilize the path loss and shadow fading models from the 3GPP Urban Microcell model in ([35], Tab,7.4.1-1). Moreover, we adopt the system configuration of [36], in which the bandwidth is B=20MHz and the maximum UE transmit power is ρ=100mW. Also, the coherence blocks contain $\tau_{c}=200$ channel, which could be achieved through a 2ms coherence time and a 100kHz coherence bandwidth. Furthermore, we assume that the channel is utilized for uplink pilot transmission for a duration of $\tau_{p}=10$, and the remainder is used for data transmission. Table 2 shows the simulation parameters used in this paper.

### 5.2. Performance Analysis

In this section, we analyze the SE performance of the proposed AP-UE association algorithm using the results derived from Equation (25). To compare the SE performance of the proposed AP-UE association algorithm, we conduct performance comparisons with the “scalable algorithm”, “structured algorithm”, and “grant algorithm” association schemes proposed in [12], [21], and [22], respectively. Additionally, a comparison with theoretical performance was also conducted assuming perfect CSI.

To provide a comprehensive performance evaluation, we compare our proposed AP-UE association algorithm with an adapted version of the baseline solution from [12]. This adapted baseline assumes that UEs sharing the same pilot sequence do not utilize orthogonal time/frequency resources, aligning with our focus on scenarios where UEs share the same time-frequency resources.

**Figure 2:**
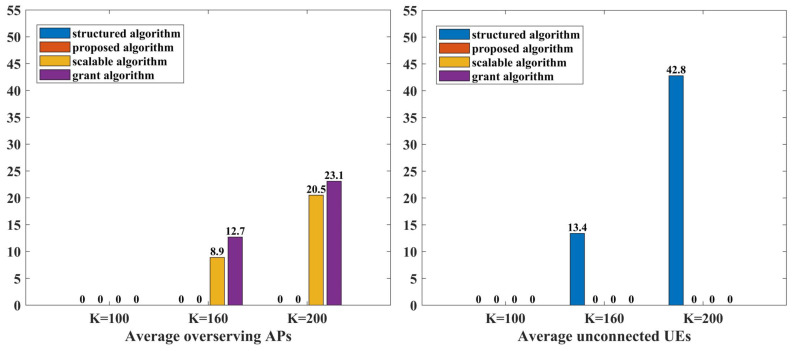
Average number of overserving APs and unconnected UEs for each algorithm.

The algorithms proposed in [12,22] ensured the connectivity of all UEs. However, as the number of UEs in the system increases, the number of overserving APs also increases, leading to potential severe pilot contamination. For instance, in Figure 2, the scalable algorithm and the grant algorithm show no overserving APs for K=100, but the average number of overserving APs rises to 17.8% and 25.4% for K=160, and further to 41% and 46.2% for K=200, respectively. In contrast, the proposed algorithm and the structured algorithm effectively prevent overserving APs across all tested scenarios. Furthermore, the algorithm proposed in [21] limits the maximum number of UEs that an AP can support to prevent strong pilot contamination, but this can lead to connectivity issues for UEs with small LSFCs. Specifically, the structured algorithm shows an average of 8.3% unconnected UEs at K=160 and 21.4% at K=200. These results indicate that while the algorithms in [12,22] cannot avoid the effects of strong pilot contamination, the algorithm in [21] reveals some UEs may not be connected. Overall, the proposed algorithm demonstrates superior performance by ensuring no overserving APs and maintaining connectivity for all UEs in massive access scenarios.

**Figure 3:**
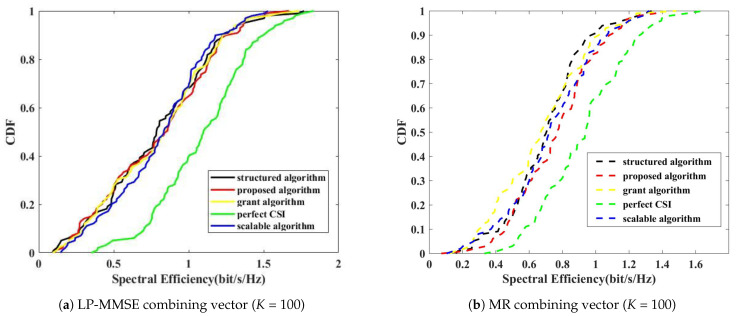

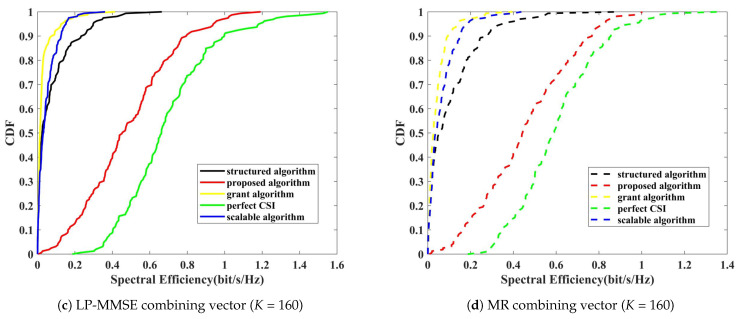
Spectral efficiency performance results based on the number of users and combining vectors.

Figure 3 presents the SE performance comparison based on the number of UEs for b=3. The performance comparison utilizes combining techniques such as LP-MMSE and MR combining.

In Figure 3, all techniques show similar performance for K=100, as there are no overserving APs or unconnected UEs affecting the outcomes. The LP-MMSE combiner performs better than the MR combiner across all scenarios. However, when the number of UEs increases to K=160, the performance of both the grant algorithm and the scalable algorithm degrades significantly due to an increase in the number of overserving APs. Specifically, both the LP-MMSE and MR combiners in the proposed algorithm only experience a performance reduction of approximately 52% and 37%, respectively, compared to their performance at K=100. In contrast, the structured algorithm, which suffers from unconnected UEs, sees a 93% performance decrease. The scalable algorithm, with fewer overserving APs, experiences a 95% decrease, while the grant algorithm, with the highest occurrence of overserving APs, suffers a 97% decline in performance. This stark contrast highlights the impact of pilot contamination on SE performance for the other algorithms, particularly in environments with dense UE deployment. Conversely, the proposed algorithm maintains its effectiveness because it does not have overserving APs or unconnected UEs, thus confirming its robustness in handling massive access scenarios.

**Figure 4:**
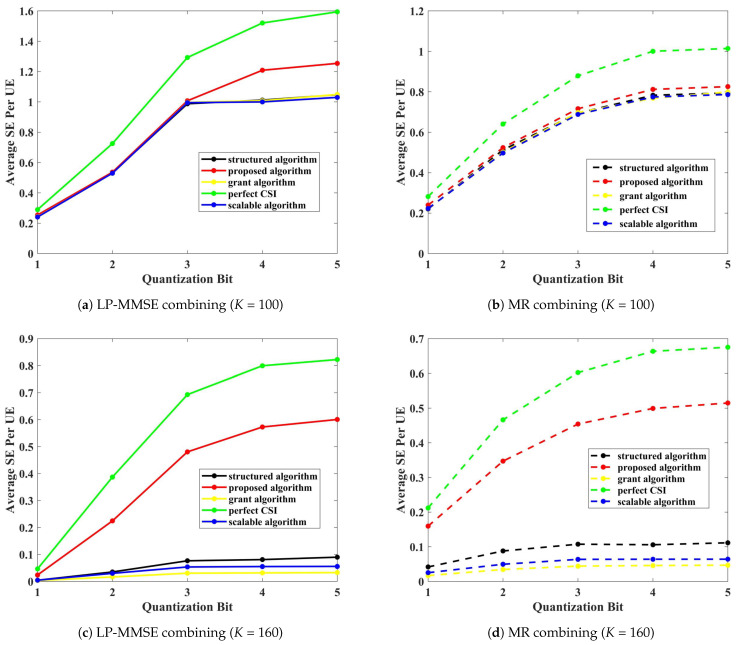
Average SE based on the number of users, combining techniques, and quantization bit.

Figure 4 compares the average SE per UE performance based on the number of users and quantization bits. Specifically, when evaluating the 3-bit to 5-bit quantization interval, the proposed algorithm experiences a performance reduction of approximately 53% for the LP-MMSE combiner and about 36% for the MR combiner as the number of UEs increases from K=100 to K=160. In contrast, the structured algorithm, which suffers from unconnected UEs, shows a performance drop of approximately 93% within the same quantization range. The scalable algorithm, which encounters fewer overserving APs, exhibits a 95% decline, while the grant algorithm, with a higher occurrence of overserving APs, sees a 97% decrease. This analysis highlights that the proposed algorithm maintains superior performance under increased UE load, effectively managing overserving APs and unconnected UEs, unlike other algorithms that experience significant SE degradation due to pilot contamination and resource allocation inefficiencies.

When quantizing with 3 bits and 5 bits, the average SE performance per UE can be compared. With 100 UEs, the LP-MMSE combiner shows 20% better performance with 5 bits compared to 3 bits, while the MR combiner shows a 10% improvement. With 160 UEs, the LP-MMSE combiner performs 10% better with 5 bits compared to 3 bits, and the MR combiner shows a 5% improvement. This shows that as the number of UEs increases, the gap in average SE performance between 3-bit and 5-bit quantization decreases.

As the quantization bit increases, an improvement in the average SE performance per UE is observed. However, this also leads to an increase in the required fronthaul capacity and power consumption.

**Figure 5:**
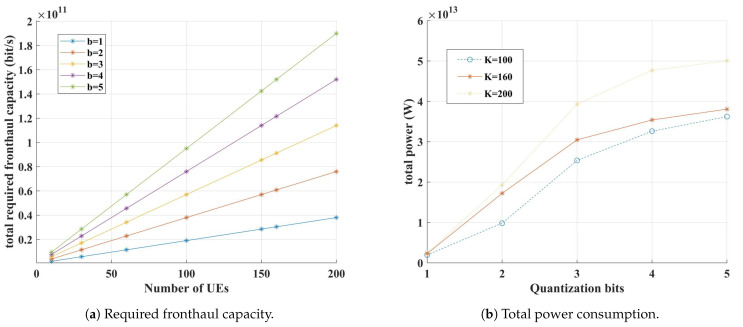
Total required fronthaul capacity and total power consumption based on the quantization bit.

Figure 5a shows the total fronthaul capacity required based on the quantization bit and the number of UEs, calculated using Equation (56) from [26]. When the number of UEs increases from 100 to 160 with 3-bit quantization, the required fronthaul capacity increases by 60%. Additionally, when comparing 3-bit to 5-bit quantization with 100 UEs, the required fronthaul capacity increases by approximately 66%. On the other hand, when using 5-bit quantization with 100 UEs, the required fronthaul capacity is about 4% higher than with 3-bit quantization and 160 UEs. This shows that 3-bit quantization allows for accommodating more UEs than 5-bit quantization. Furthermore, with reduced fronthaul capacity requirements, the CPU’s fronthaul load also decreases compared to 5-bit quantization. Therefore, in massive access scenarios with a large number of UEs, reducing the quantization bits can decrease the fronthaul load while allowing the network to support many UEs.

Figure 5b shows the total power consumption based on the quantization bit, calculated using Equation (28) from [27]. When the number of UEs is 100, 5-bit quantization consumes about 44% more power than 3-bit quantization. Additionally, when the number of UEs is 160, 5-bit quantization consumes about 26% more power than 3-bit quantization. Comparing this to the average SE performance, with 100 UEs, 5-bit quantization shows a 20% increase in average SE performance over 3-bit quantization, but the power consumption increases by 44%. With 160 UEs, the average SE performance increases by 10%, but the power consumption increases by 26%. This indicates that in environments with a large number of UEs, the difference in average SE performance between 3-bit and 5-bit quantization is not significant, making 3-bit quantization more effective in reducing power consumption.

## 6. Conclusions

In this paper, we propose an AP-UE association algorithm for massive access in a user-centric cell-free massive MIMO system employing low-resolution ADCs. Current user-centric cell-free massive MIMO systems typically assume infinite fronthaul capacity and the use of high-resolution ADCs by APs. However, in reality, fronthaul capacity is not infinite. Additionally, in massive access scenarios, there is a need for methods to reduce the required fronthaul capacity. Hence, this study introduces the adoption of low-resolution ADCs and approximates the quantization noise associated with these ADCs using the AQNM model. Additionally, we derive a closed-form expression for the achievable rate.

Furthermore, we propose an AP-UE association algorithm based on heuristic approaches that ensure performance even in massive access scenarios. The existing association methods do not consider the scenario of massive access in which the number of UEs far exceeds the number of APs, and they perform association only from the perspective of APs or UEs. However, the proposed association method considers the scenario of massive access in the user-centric cell-free massive MIMO system with low-resolution ADCs. We compare its performance with existing methods in terms of SE per UE. Through performance comparison in terms of SE per UE, the proposed algorithm shows better performance than existing algorithms. Moreover, at K=160, while existing algorithms fail to guarantee performance, the proposed algorithm ensures the performance.

Additionally, we performed simulations to require fronthaul capacity and power consumption when utilizing low-resolution ADCs. Our findings indicate that reducing quantization bits in massive access scenarios reduces fronthaul load and enhances the capability to accommodate more UEs. Furthermore, as the number of UEs increases, the performance gap in average SE between 3-bit and 5-bit quantization diminishes. Specifically, in terms of power efficiency, our simulations confirmed that 3-bit quantization outperforms 5-bit quantization in massive access scenarios.

Future research needs to more accurately predict the quantization model that exhibits nonlinear characteristics due to low-resolution ADCs. While the AQNM model provides a useful framework for approximating quantization noise, it has limitations, particularly in accurately capturing the nonlinear characteristics introduced by low-resolution ADCs. The model assumes a linear approximation which may not fully account for the complexity of signal distortions in practical scenarios. Future research needs to develop more accurate quantization models that can predict these nonlinear effects with greater precision. Additionally, accurate channel estimation will also be necessary. Deep learning could enable more precise predictions of these nonlinear characteristics and also improve the accuracy of channel estimation. This would significantly impact performance improvement. 

## Figures and Tables

**Table 1:** The distortion parameter of the additive quantization noise model (derived from [31]).

*b*	1	2	3	4	5
$\rho_{\alpha }$	0.3634	0.1175	0.03454	0.009497	0.002499
α	0.6366	0.8825	0.96546	0.990503	0.997501

**Table 2:** Simulation parameters.

Parameters	Value
area	0.5km×0.5km
Number of APs	50
Number of Antennas for AP	4
Number of Antennas for UE	1
Bandwidth	20MHz
UE transmit power (ρ)	100mW
Coherence block $\left(\tau_{c}\right)$	200 sample
Pilot length $\left(\tau_{p}\right)$	10 sample

## Data Availability

No new data were created or analyzed in this study. Data sharing is not applicable to this article.
